# Association of optic disc pallor and RNFL thickness with cerebral small vessel disease in the PREVENT‐Dementia study

**DOI:** 10.1002/dad2.12633

**Published:** 2024-08-08

**Authors:** Samuel Gibbon, Audrey Low, Charlene Hamid, Megan Reid‐Schachter, Graciela Muniz‐Terrera, Craig W. Ritchie, Emanuele Trucco, Baljean Dhillon, John T. O'Brien, Thomas J. MacGillivray

**Affiliations:** ^1^ Centre for Clinical Brain Sciences Chancellor's Building Edinburgh UK; ^2^ Robert O Curle Ophthalmology Suite Institute for Regeneration and Repair Edinburgh UK; ^3^ Department of Psychiatry School of Clinical Medicine University of Cambridge Cambridge UK; ^4^ Heritage College of Osteopathic Medicine Ohio University Athens Campus Athens Ohio USA; ^5^ VAMPIRE project, Computing (SSEN) University of Dundee Queen Mother Building Dundee UK; ^6^ Princess Alexandra Eye Pavilion Edinburgh UK; ^7^ Edinburgh Imaging The Queen's Medical Research Institute University of Edinburgh Edinburgh UK

**Keywords:** cerebral small vessel disease, dementia, magnetic resonance imaging, optic disc pallor, retina

## Abstract

**INTRODUCTION:**

We tested associations between two retinal measures (optic disc pallor, peripapillary retinal nerve fiber layer [pRNFL] thickness) and four magnetic resonance imaging markers of cerebral small vessel disease (SVD; lacunes, microbleeds, white matter hyperintensities, and enlarged perivascular spaces [ePVSs]).

**METHODS:**

We used PallorMetrics to quantify optic disc pallor from fundus photographs, and pRNFL thickness from optical coherence tomography scans. Linear and logistic regression assessed relationships between retinal measures and SVD markers. Participants (*N* = 108, mean age 51.6) were from the PREVENT Dementia study.

**RESULTS:**

Global optic disc pallor was linked to ePVSs in the basal ganglia in both left (*β* = 0.12, standard error [SE] = 0.05, *P* < 0.05) and right eyes (*β* = 0.13, SE = 0.05, *P* < 0.05). Associations were also noted in different disc sectors. No pRNFL associations with SVD markers were found.

**DISCUSSION:**

Optic disc pallor correlated with ePVSs in the basal ganglia, suggesting retinal examination may be a useful method to study brain health changes related to SVD.

**Highlights:**

- Optic disc pallor is linked to enlarged perivascular spaces in basal ganglia.
- There is no association between peripapillary retinal nerve fiber layer thickness and cerebral small vessel disease markers.
- Optic disc examination could provide insights into brain health.
- The sample included 108 midlife adults from the PREVENT Dementia study.

## 1 INTRODUCTION

Cerebral small vessel disease (SVD) is an age‐related condition affecting the arterioles, venules, and capillaries of the central nervous system, and is a major cause of stroke, dementia, and cognitive decline.^1^, ^2^, ^3^ SVD can be detected in the brain using magnetic resonance imaging (MRI). The most widely recognized MRI‐visible features of SVD include lacunes, microbleeds, white matter hyperintensities (WMHs), and enlarged perivascular spaces (ePVSs).^1^, ^2^, ^3^, ^4^ Due to high cost and low availability, MRI is not suited for mass screening. Instead, the retina, owing to its homology with the brain,^5^ and the relative ease with which images can be acquired, has become a target for biomarker discovery in SVD.^6^, ^7^ One such promising imaging modality is optical coherence tomography (OCT), which can capture the peripapillary retinal nerve fiber layer (pRNFL), a thinning of which represents a loss of retinal ganglion cells/axons and is associated with SVD^8^, ^9^, ^10^ and dementia.^11^ However, the resolution of current OCT devices and the repeatability of scanning and analysis may not have sufficient sensitivity to discern subtle changes in the preclinical stages of dementia.^12^, ^13^ Recently, we developed PallorMetrics,^14^ an alternative for studying pRNFL based on measuring optic disc pallor in fundus photographs, on the premise that a pale disc may indicate loss or degeneration of the pRNFL. In this study, we explore the associations between both pRNFL thickness and continuous measures of optic disc pallor with four MRI‐derived markers of SVD (lacunes, microbleeds, WMHs, ePVSs).

## 2 METHODS

### 2.1 Participants and image capture

The PREVENT Dementia study protocol has been described elsewhere.^15^ Briefly, 700 participants aged 40 to 59, enriched for family history of dementia (targeted 50% with family history), were recruited from five sites in the UK and Ireland. Brain MRI was performed on 648 participants using 3T Siemens Scanners (Verio, Skrya, and Prisma); sequence and acquisition parameters have been described in detail previously.^16^, ^17^ Briefly, for the 3D T1‐weighted (T1w) magnetization‐prepared rapid acquisition gradient echo images, there were 160 slices, a repetition time (TR) of 2300 ms, an echo time (TE) of 2.98 ms, a 9° flip angle, and a 1 × 1 × 1 mm^3^ voxel size. The T2‐weighted (T2w) scans involved 32 slices, a TR of 1500 ms, a TE of 80 ms, a 150° flip angle, and a voxel size of 0.69 × 0.69 × 4 mm^3^. For fluid‐attenuated inversion recovery (FLAIR) images, there were 27 slices, a TR of 9000 ms, a TE of 94 ms, a 150° flip angle, and a voxel size of 0.43 × 0.43 × 4 mm^3^. Susceptibility‐weighted imaging (SWI) scans used 72 slices, a TR of 28 ms, a TE of 20 ms, a 15° flip angle, and a voxel size of 0.72 × 0.72 × 1.2 mm^3^.

**RESEARCH IN CONTEXT**

1. **Systematic review**: We reviewed the literature on the relationship between optic disc pallor, retinal nerve fiber layer thickness, and markers of cerebral small vessel disease (SVD). While retinal health is known to reflect brain health, few studies have specifically linked optic disc pallor to SVD markers.
2. **Interpretation**: We found a significant association between increased optic disc pallor and enlarged perivascular spaces in the basal ganglia. This suggests that optic disc pallor could be an early indicator of SVD, supporting the use of retinal imaging as a non‐invasive method for detecting brain health issues like stroke and dementia.
3. **Future directions**: Future research should confirm these associations in larger, more diverse populations, investigate the mechanisms linking optic disc pallor and SVD, and evaluate retinal imaging as an adjunctive tool for assessing brain health. Longitudinal studies are needed to see if changes in optic disc pallor can predict SVD progression and subsequent cognitive decline.

**FIGURE 1:**
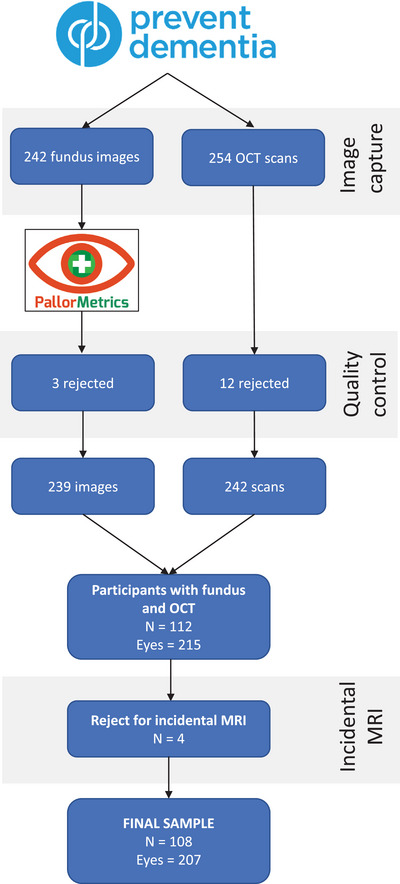
Participant selection flowchart. OCT, optical coherence tomography

Retinal imaging was a substudy conducted at the Edinburgh site only.^18^ Exclusion criteria included participants with current or previous ocular disease such as glaucoma, macular degeneration, diabetic retinopathy, uveitis, vitreous hemorrhage, ischemic optic neuropathy, optic neuritis or other optic nerve diseases, or those who had undergone previous ocular surgery such as cataract surgery or retinal surgery. Retinal fundus photographs were captured using a non‐mydriatic camera (CR‐DGi; Canon USA, Inc.), with a 45° field of view. The protocol was to capture the posterior pole, with an image centered halfway between the macula and the optic disc. OCT was captured with a Heidelberg SPECTRALIS machine (Heidelberg Engineering) using the N‐site peripapillary module set to high speed (1536 A‐scans), with a target Automatic Real Time‐function (ART) of 100. Participants provided written informed consent, and the study was carried out in compliance with the Declaration of Helsinki. Due to reasons including device/computer error, imaging room availability, poor cooperation, small pupil size, and participant feeling unwell, it was not always possible to capture both fundus images and OCT of each eye from each participant (Figure 1, top bar). After quality control (detailed in the section “Quality control”) and the removal of four participants with incidental MRI findings (previously screened^17^), concurrent fundus images and OCT scans were available for 108 participants (207 eyes; Figure 1).

### 2.2 SVD quantification

SVD quantification for the PREVENT Dementia cohort has been described elsewhere.^16^, ^17^ Briefly, ePVSs were assessed separately in the basal ganglia and centrum semiovale in T2w MRI using a validated rating scale.^19^ Scores ranged from 0 to 4 based on the number of lesions: 0 (none), 1 (1–10), 2 (11–20), 3 (21–30), 4 (> 40). ePVSs in the midbrain were dichotomized as 0 (absent) or 1 (present). Cerebral microbleeds were assessed separately in the lobar (centrum semiovale) and deep (basal ganglia, thalamus) regions on SWI MRI, and cross‐checked against T1w and T2w MRI using a validated scale.^20^ Scores were binarized as 0 (absent) or 1 (present in one or both regions). Similarly, lacunes were assessed in the same regions but in T1w, T2w, and FLAIR MRI^4^ and binarized in the same manner. Each SVD marker was evaluated by a single rater and a subset (20%) by a second rater. Inter‐rater reliability (Cohen kappa) was good: 0.74 for cerebral microbleeds, 0.92 for lacunes, and 0.85 for ePVSs.

WMH total lesion volume was measured according to a protocol described elsewhere.^16^ Briefly, in a semi‐automated manner, gray matter, white matter, and cerebrospinal fluid were segmented from T1w images to create a binary mask. This mask was then registered to, and subtracted from, the corresponding FLAIR images. All WMH lesion masks were visually inspected and manually corrected for misclassifications. WMH volume was extracted from lesion masks, providing global WMH volume and regional volumes (periventricular WMH, deep WMH). WMH volume was then cube‐root transformed.

### 2.3 Optic disc pallor quantification

**FIGURE 2:**
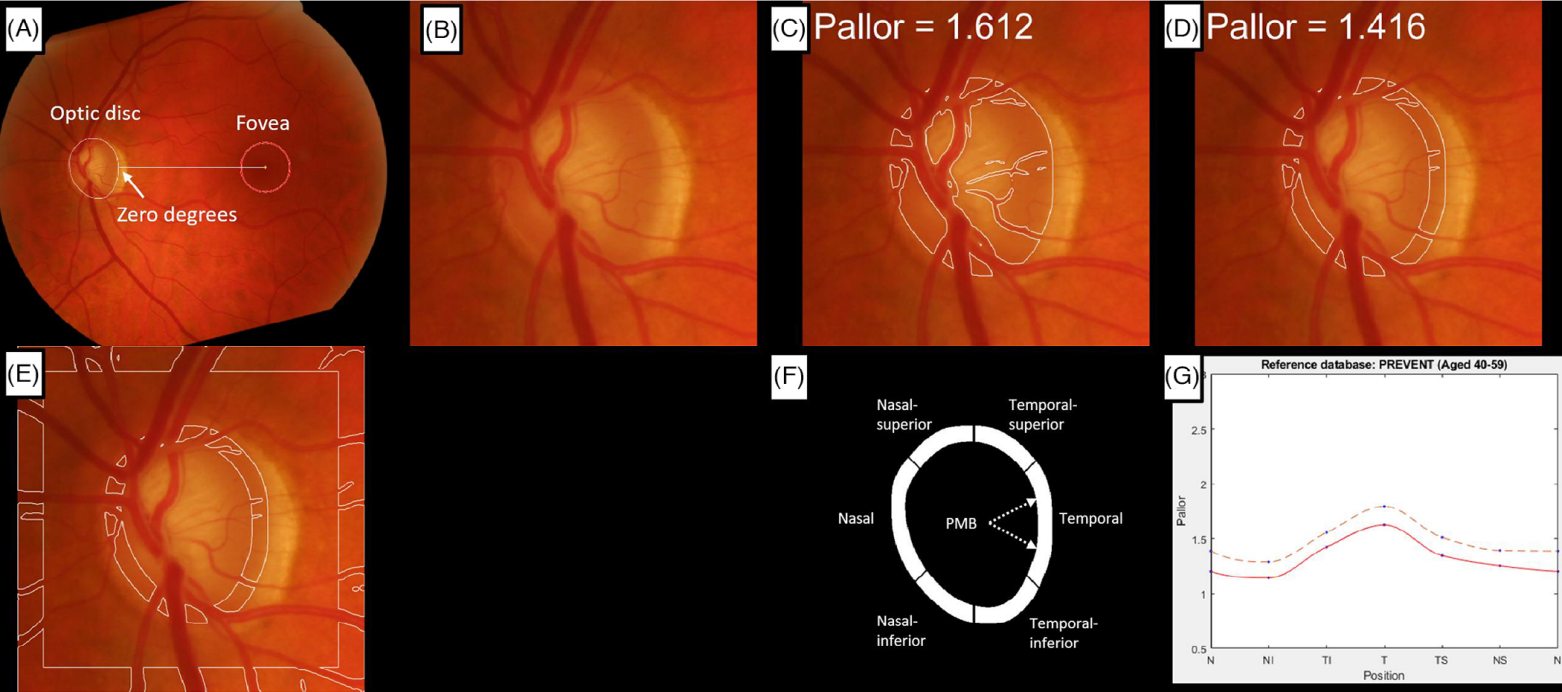
Visual output of the pallor software for a single image. A, Full image rotated along the optic disc‐fovea axis. B, Cropped optic disc. C, Segmented whole disc with pallor value. D, Segmented measurement region with global pallor value. E, Measurement and control region. F, Zones and alert system (region turns red if a limit is exceeded). G, Dashed line represents one standard deviation above the mean of all participants, red line is the current participant. Notes: Vessels are excluded in (C), (D), and (E)

Optic disc pallor was measured automatically in color fundus photographs using previously validated software, PallorMetrics.^14^ Briefly, a deep learning–based approach was used to localize the fovea and segment the optic disc to the inner edge of the border tissue (Figure 2A, C). A measurement region was defined to start at the disc border and extend 30 pixels inward (Figure 2D). Zones were placed over the measurement region in accordance with the Heidelberg SPECTRALIS peripapillary scan (Figure 2F). Specifically, the intersection of the fovea–optic disc axis with the measurement region was given a value of 0° (Figure 2A). The temporal zone extended from –45° to 45°, the temporal superior from –45° to –90°, and so on. The papillomacular bundle (PMB; a thick bundle of axons originating in the macula that is responsible for central, sharp vision) is a special case of the temporal zone, extending from –15° to 15°. Finally, pallor was measured as a function of red/green light reflectance in each zone with respect to a control region (Figure 2E). The resulting pallor values are dimensionless, and the disc area is in pixels.

### 2.4 Quality control

Of 242 fundus images presented to the Pallor software, three were subsequently rejected due to: segmentation error (two images) and abnormal disc presentation (one image; Figure S1 in supporting information). Of 254 OCT scans, 12 were rejected due to one or more of improper centering (four scans), clipping (four scans), high myopia (≥ –5D; two scans), poor segmentation (three scans), poor illumination (one scan), and signs of pathology (five scans). Pathologies included excessive peripapillary atrophy, excessively tilted discs, and epiretinal membrane. OCT quality control was conducted by an ophthalmic imager and analyst (author C.H.) via manual inspection of the scans through the Heidelberg Engineering HeyEx device software platform.

### 2.5 Statistical analysis

To minimize false positives, our approach was 2‐fold. First, we tested for associations between each SVD marker and global pallor and global pRNFL thickness. When a significant association was found, we then proceeded to test for associations with all eight zonal measures of pallor and pRNFL (seven zones + nasal/temporal ratio). Second, we tested the left and right eyes independently and only considered a finding genuine if it was significant in both eyes. We treated ePVS as a continuous variable—with a range of 0–4 (we did not have access to the full count). We used linear regression for continuous SVD markers (WMH, ePVS), and logistic regression for binary markers (lacunes, microbleeds). Our decision to treat ePVS as continuous was motivated in part due to the limitations of ordinal logistic regression (the main alternative to linear modeling for this case), which can be difficult to interpret and assumes proportional odds, in part to maintain alignment with previous work,^16^, ^17^ which also treats ePVS as continuous, and in part to make it easier for readers to compare coefficients across multiple SVD markers. Nonetheless, we acknowledge that linear models may have power limitations in the current context. All models were adjusted for age, sex, history of hypertension, fasting cholesterol, body mass index, and smoking status (current, ex, never). Pallor measurements may be affected by differences in retinal magnification as a result of varying axial length and disc area.^14^ Given that disc area is a correlate of axial length,^21^ we opted to control for disc area in our models. We further acknowledge that mixed effect models may have been preferable as they can account for the nested structure of eyes within participants and allow the use of all datapoints in a single model; however, the small sample size and a relatively large number of covariates precluded their use. Only three participants were current smokers, so we collapsed “current” with “ex,” and only three participants had diabetes, so we did not include diabetes as a covariate. All continuous variables were standardized. Rows containing missing values (detailed in Table 1) were excluded at the point of analysis. Analyses were conducted in R (version 4.2.1; www.R‐project.org).

**TABLE 1:** Demographics and study variables by zone and eye.

	Eye		
	*Left*	*Right*	*Overall*
*N* (participants)			108
*N* (eyes)	102	105	207
**Age**	51.6 (5.6)	51.6 (5.5)	51.6 (5.5)
**Sex (female)**	64 (62.7%)	66 (62.9%)	130 (62.8%)
**Hypertension diagnosis**	8 (7.8%)	8 (7.6%)	16 (7.7%)
**BMI**	28.7 (5.8)	28.5 (5.9)	28.6 (5.8)
**Cholesterol**	5.47 (1.1)	5.42 (1.0)	5.44 (1.0)
**Smoking**			
Current	2 (2.0%)	2 (1.9%)	4 (1.9%)
Ex	36 (35.3%)	37 (35.2%)	73 (35.3%)
Non	63 (61.8%)	65 (61.9%)	128 (61.8%)
**White matter hyperintensities**			
Total	2.72 (3.2)	2.7 (3.2)	2.71 (3.2)
Deep	1 (1.26)	0.99 (1.24)	0.99 (1.24)
Periventricular	1.72 (2.2)	1.71 (2.2)	1.72 (2.2)
**Enlarged perivascular spaces**			
Count			
Basal ganglia	12 (11.8%)	11 (10.5%)	23 (11.1%)
0			
1	76 (74.5%)	79 (75.2%)	155 (74.9%)
2	12 (11.8%)	13 (12.4%)	25 (12.1%)
Centrum semiovale			
0	12 (11.8%)	11 (10.5%)	23 (11.1%)
1	62 (60.8%)	63 (60.0%)	125 (60.4%)
2	17 (16.7%)	20 (19.0%)	37 (17.9%)
3	8 (7.8%)	8 (7.6%)	16 (7.7%)
4	1 (1.0%)	1 (1.0%)	2 (1.0%)
Midbrain (present)	61 (59.8%)	64 (61.0%)	125 (60.4%)
**Lacunes (present)**	13 (12.7%)	15 (14.3%)	28 (13.5%)
**Microbleeds (present)**	21 (20.6%)	22 (21.0%)	43 (20.8%)
**Optic disc pallor**			
Global	1.40 (0.18)	1.34 (0.17)	1.37 (0.18)
Temporal	1.62 (0.24)	1.55 (0.23)	1.58 (0.24)
Temporal–inferior	1.42 (0.20)	1.34 (0.19)	1.38 (0.20)
Nasal–inferior	1.16 (0.14)	1.12 (0.14)	1.14 (0.14)
Nasal	1.24 (0.16)	1.18 (0.16)	1.21 (0.16)
Nasal–superior	1.27 (0.16)	1.20 (0.15)	1.23 (0.16)
Temporal–superior	1.36 (0.19)	1.31 (0.18)	1.33 (0.18)
PMB	1.68 (0.25)	1.60 (0.24)	1.64 (0.25)
Nasal/temporal ratio	0.769 (0.06)	0.771 (0.08)	0.770 (0.07)
**RNFL thickness**			
Global	98.2 (8.2)	98.3 (8.1)	98.3 (8.1)
Temporal	69.7 (13.6)	74.5 (13.2)	72.1 (13.6)
Temporal–inferior	140 (19.5)	141 (22.9)	141 (21.2)
Nasal–inferior	114 (22.1)	112 (22.5)	113 (22.3)
Nasal	74.3 (16.3)	76.3 (19.0)	75.3 (17.7)
Nasal–superior	111 (19.3)	98.7 (18.8)	105 (20.0)
Temporal–superior	135 (17.4)	135 (17.0)	135 (17.1)
PMB	54.2 (13.2)	56.1 (9.1)	55.1 (11.3)
Nasal/temporal ratio	1.11 (0.3)	1.06 (0.4)	1.08 (0.3)
**Retinal covariates**			
Disc area	75,700 (15,700)	73,600 (14,200)	74,600 (14,900)

*Notes*: All values are mean (standard deviation) or N (%). Missing data (variable, number of participants): Hypertension diagnosis (2), body mass index (4), cholesterol (8), smoking status (2), diabetes (2), white matter hyperintensities (4), enlarged perivascular spaces (4), lacunes (4), microbleeds (6), global pRNFL (2), nasal pRNFL (2), nasal–superior pRNFL (2), papillomacular bundle RNFL (4), nasal/temporal ratio pRNFL (6).

Abbreviations: BMI, body mass index; NT, nasal/temporal; PMB, papillomacular bundle; pRNFL, peripapillary retinal nerve fiber layer thickness.

### 2.6 Data availability

Data are available upon reasonable request from the PREVENT‐Dementia study team https://preventdementia.co.uk/.

## 3 RESULTS

The final sample had a mean age of 51.6 years (standard deviation [SD] = 5.5) and contained a higher proportion of females (62.8%). Scores from SVD markers were similar between the left and right eyes. Demographics and study variables are summarized in Table 1, and visualizations from the pallor software for a single fundus image are presented in Figure 2.

**FIGURE 3:**
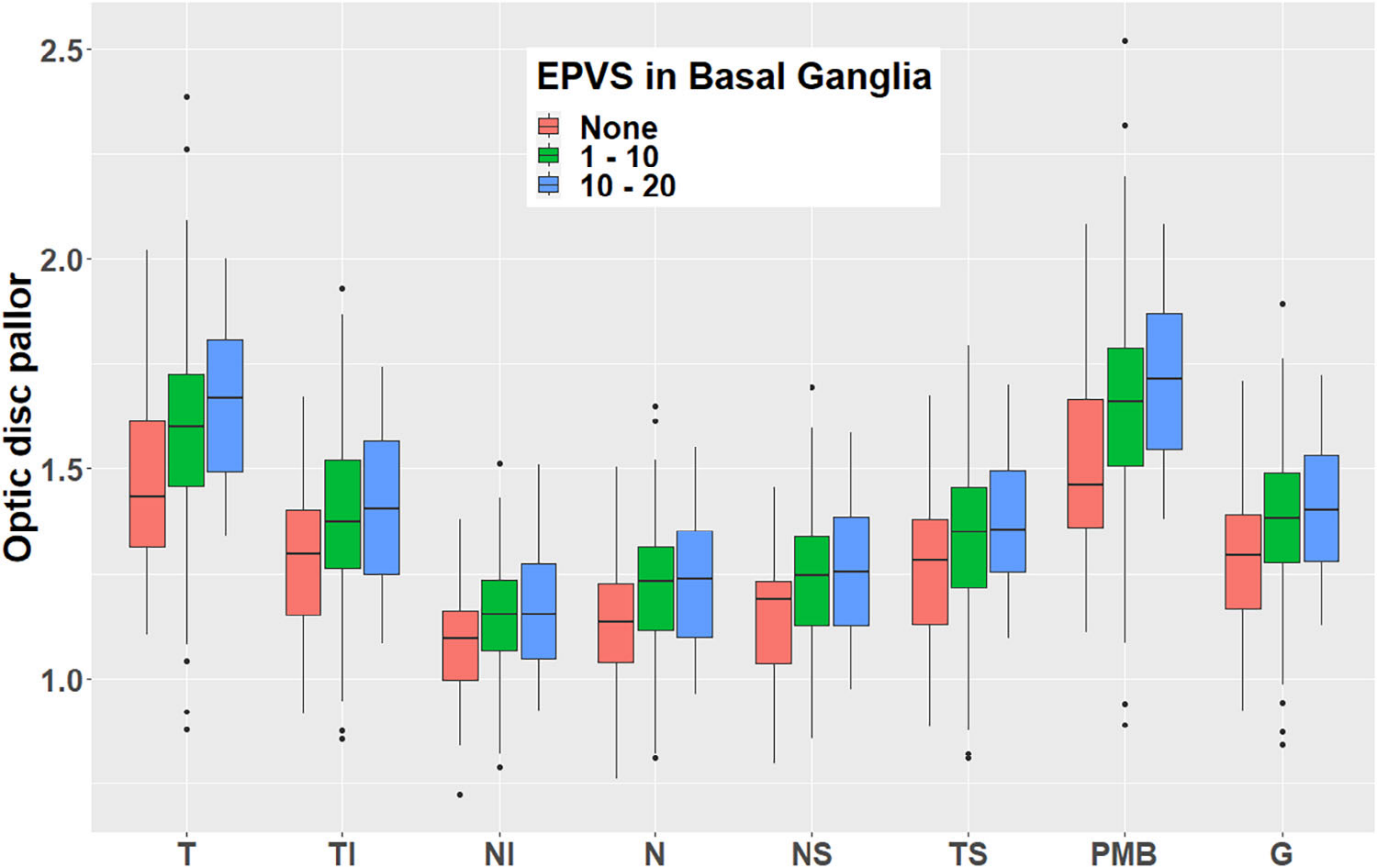
Grouped box plot showing the relationship between the number of EPVSs in the basal ganglia and optic disc pallor by zone. Data aggregated from both eyes. EPVSs, enlarged perivascular spaces; G, global; I, inferior; N, nasal; S, superior; T, temporal

We observed evidence of significant associations between ePVSs in the basal ganglia and global pallor in both the left eye (standardized β; *β* = 0.12 [standard error (S)E = 0.05], *P* < 0.05), and the right eye (*β* = 0.13 [SE = 0.05], *P* < 0.05). By zone, we found associations between ePVSs in the basal ganglia and disc pallor in the temporal (*β* = 0.11 [SE = 0.05], *P* < 0.05), temporal–inferior (*β* = 0.11 [SE = 0.05], *P* < 0.05), nasal–inferior (*β* = 0.11 [SE = 0.05], *P* < 0.05), nasal (*β* = 0.12 [SE = 0.05], *P* < 0.05), nasal–superior (*β* = 0.13 [SE = 0.05], *P* < 0.05) and PMB (*β* = 0.11 [SE = 0.05], *P* < 0.05) zones in the left eye, and the temporal (*β* = 0.13 [SE = 0.05], *P* < 0.05], temporal–inferior (*β* = 0.15 [SE = 0.05], *P* < 0.01), nasal (*β* = 0.10 [SE = 0.05], *P* < 0.05), nasal–superior (*β* = 0.13 [SE = 0.05], *P* < 0.05), and PMB (*β* = 0.13 [SE = 0.05], *P* < 0.05) zones in the right eye. There was no evidence of pallor being associated with any other MRI markers. Similarly, we observed no evidence of associations between pRNFL thickness and any of the MRI markers. Results are summarized in Table 2 and presented graphically in Figure 3.

**TABLE 2:** Linear (WMH, ePVS) and logistic (lacunes, microbleeds) regression results for each eye.

			Eye			
			*Left*		*Right*	
	Retinal zone	Imaging modality	*β (SE)/odds ratio (CI)*	*P value*	*β (SE)/odds ratio (CI)*	*P value*
**White matter hyperintensities**						
Total	*Global*	Fundus	0.10 (0.11)	0.377	0.05 (0.11)	0.648
		OCT	–0.08 (0.10)	0.453	–0.03 (0.10)	0.781
Periventricular	*Global*	Fundus	0.06 (0.11)	0.596	0.03 (0.11)	0.803
		OCT	–0.07 (0.10)	0.576	–0.02 (0.10)	0.866
Deep	*Global*	Fundus	0.14 (0.11)	0.198	0.08 (0.11)	0.482
		OCT	–0.08 (0.11)	0.440	–0.04 (0.10)	0.691
**Enlarged perivascular spaces**						
Basal ganglia	*Global*	Fundus	0.12 (0.05)	**0.020** ^*^	0.13 (0.05)	**0.141** ^*^
		OCT	–0.04 (0.05)	0.408	–0.03 (0.05)	0.538
	*Temporal*	Fundus	0.11 (0.05)	**0.022** ^*^	0.13 (0.05)	**0.013** ^*^
	*Temporal–Inferior*	Fundus	0.11 (0.05)	**0.015** ^*^	0.15 (0.05)	**0.008** ^**^
	*Nasal–Inferior*	Fundus	0.11 (0.05)	**0.026** ^*^	0.09 (0.05)	0.082
	*Nasal*	Fundus	0.12 (0.05)	**0.016** ^*^	0.10 (0.05)	**0.042** ^*^
	*Nasal–Superior*	Fundus	0.13 (0.05)	**0.013** ^*^	0.13 (0.05)	**0.013** ^*^
	*Temporal–Superior*	Fundus	0.09 (0.05)	0.067	0.10 (0.05)	0.064
	*PMB*	Fundus	0.11 (0.05)	**0.023** ^*^	0.13 (0.05)	**0.014** ^*^
	*NT ratio*	Fundus	–0.01 (0.05)	0.783	–0.04 (0.04)	0.379
Midbrain	*Global*	Fundus	1.34 (0.82–2.25)	0.249	1.04 (0.61–1.75)	0.884
		OCT	0.82 (0.51–1.30)	0.400	0.90 (0.56–1.43)	0.650
Centrum sem.	*Global*	Fundus	0.01 (0.09)	0.881	0.06 (0.09)	0.522
		OCT	0.03 (0.09)	0.761	0.03 (0.08)	0.698
**Lacunes**	*Global*	Fundus	1.49 (0.77–3.05)	0.247	1.20 (0.63–2.36)	0.576
		OCT	1.29 (0.68–2.56)	0.445	1.44 (0.79–2.72)	0.245
**Microbleeds**	*Global*	Fundus	0.87 (0.47–1.56)	0.638	0.87 (0.48–1.54)	0.624
		OCT	1.21 (0.70–2.16)	0.501	1.37 (0.82–2.39)	0.248

*Note*: All models were adjusted for age, sex, diagnosed hypertension, cholesterol, body mass index, and smoking status. Pallor/fundus models were additionally adjusted for disc size.

Abbreviations: CI, confidence interval; ePVS, enlarged perivascular space; NT, nasal/temporal; OCT, optical coherence tomography; PMB, papillomacular bundle; SE, standard error of the mean; WMH, white matter hyperintensity.

* *P* < 0.05

** *P* < 0.01.

## 4 DISCUSSION

Independent of covariates, we found that disc pallor, but not pRNFL thickness, was significantly associated with ePVSs in the basal ganglia globally, and in several zones. These associations were found independently in the left and right eyes. ePVSs are commonly observed in SVD pathologies^22^, ^23^ and have been associated with increased age and cardiovascular risk factors,^24^ inflammation,^25^ blood–brain barrier dysfunction,^26^ cognitive decline and dementia,^27^, ^28^ and other MRI features of SVD,^23^, ^24^ yet they are difficult and costly to measure. Here, we provide evidence that increasing paleness of the optic disc, measured automatically in conventional retinal fundus images, is associated with ePVSs in the brain.

Previous research has established a connection between MRI‐derived markers of SVD and parameters measured from fundus images showing the retinal vasculature.^7^, ^29^, ^30^, ^31^, ^32^, ^33^ For example, studies have discovered that ePVSs are associated with changes in retinal vessel thickness^32^, ^33^ and reduced fractal dimension.^33^ However, there has been less focus on investigating the relationship between SVD and neuronal retinal measures. To the best of our knowledge, no other studies have explored the relationship between SVD and optic disc appearance in terms of its color or pallor. However, a limited body of work has linked SVD with pRNFL thinning in conditions such as cerebral amyloid angiopathy^8^ and cerebral autosomal dominant arteriopathy with subcortical infarcts and leukoencephalopathy (CADASIL) ^9^, ^10^ although it is worth noting that an increase in pRNFL has also been observed in CADASIL.^34^ Our research supports these findings and suggests that optic disc pallor may be more sensitive than pRNFL as an indicator of SVD.

Out of four MRI markers tested, only ePVSs in the basal ganglia were associated with an ocular measure. One reason could be statistical power. Indeed, non‐significant associations all suggested a positive association (see Table 2), so an effect may have emerged given more data. Alternatively, the observed association between increasing disc pallor and ePVSs suggests that disc pallor may be a surrogate for another pathology. Perivascular spaces/channels are the anatomical basis of the recently proposed glymphatic system,^35^, ^36^, ^37^, ^38^ a brain‐wide waste clearance system responsible for eliminating metabolites and soluble proteins from the central nervous system. Impaired glymphatic function has been associated with multiple neurological disorders,^37^, ^39^ including worsening radiological features of SVD.^40^, ^41^, ^42^

Importantly, there is a growing body of research supporting the existence of an ocular glymphatic system^38^, ^43^, ^44^, ^45^, ^46^, ^47^ that is likely continuous with the glymphatic system of the brain,^48^ extending to the visual pathway (including the retina and optic nerve). Indeed, several studies have highlighted the potential role of ocular glymphatic dysfunction in optic nerve disorders, such as glaucoma and hydrocephalus (see Kasi et al.^48^ for a review). This suggests that the ocular glymphatic system may play a crucial role in normal optic nerve function, and impairment could lead to ocular dysfunction, including the loss of retinal ganglion cells and subsequent RNFL thinning.^49^

In our study, we observed an association between brain ePVS and increasing paleness of the optic disc, which itself is believed to be driven by RNFL loss. As a result, we propose that optic disc pallor may serve as a marker of glymphatic dysfunction affecting both the brain and visual pathway. In particular, our findings align with previous research which found that impaired glymphatic function in the brain may be uniquely linked to increased ePVS in the basal ganglia, but not the centrum semiovale.^42^ Further work should investigate the effect of glymphatic function on optic disc appearance.

Although optic disc pallor is an indirect measure of pRNFL thickness, in the current study we observed associations between ePVS and disc pallor but not between ePVS and pRNFL thickness. There are two potential reasons for this apparent discrepancy. First, disc pallor can reflect not only the loss of nerve fibers but also gliosis,^50^ axonal degeneration, and other changes within the optic nerve head, which might be more directly related to the presence of ePVSs. By contrast, pRNFL measurements are specifically focused on axonal thickness and may not capture these broader changes. Furthermore, disc pallor indicates chronic, longstanding optic nerve damage, which may be more aligned with the cumulative effects of chronic SVD as evidenced by ePVSs. By contrast, pRNFL thickness changes can occur more rapidly in response to acute insults and may not reflect the chronicity of the underlying pathology as sensitively as optic disc pallor.

Strengths of this study include: (1) the concomitant use of fundus images and OCT scans to investigate multiple brain‐based SVD markers; (2) the use of novel, automated software to quantify optic disc pallor; and (3) that significant associations were found independently in the left and right eyes.

Limitations of this study include its cross‐sectional design, which precluded us from inferring causality or investigating change over time. In addition, the sample was majority female (62.8%) and White British in ethnicity, which may limit generalizability to a wider population. Finally, it should be noted that there are several other metrics available for quantifying ePVS burden including length, width, volume, sphericity, and orientation,^23^ which may have allowed for further insight into the associations between brain health and disc pallor. Future research should aim to replicate the current results in a larger and more diverse sample.

## 5 CONCLUSION

ePVSs are a hallmark feature of SVD, yet they are difficult and costly to measure. Our study suggests that increasing paleness of the optic disc may be linked to the presence of ePVSs in the basal ganglia. Our findings emphasize the value of retinal fundus imaging as a convenient and promising avenue for investigating changes in brain health associated with SVD. Further research in this direction could shed more light on the complex interplay between retinal measures and SVD, ultimately contributing to our understanding of neurodegenerative diseases.

## CONFLICT OF INTEREST STATEMENT

S. Gibbon, A. Low, C. Hamid, M. Reid‐Schachter, G. Muniz‐Terrera, C. Ritchie, E. Trucco, J. T. O'Brien, and T. J. MacGillivray report no disclosures relevant to the manuscript. B. Dhillon received an educational grant from Apellis Pharmaceuticals in 2023. Author disclosures are available in the supporting information.

## CONSENT STATEMENT

All participants provided written informed consent, and the study was carried out in compliance with the Declaration of Helsinki.

## Supporting information

Supporting Information

Supporting Information
