# Supplementary material for: Association of optic disc pallor and RNFL thickness with cerebral small vessel disease in the PREVENT‐Dementia study
Source: Alzheimers Dement (Amst). 2024 Aug 8;16(3):e12633. doi: 10.1002/dad2.12633 (PMC11307169; doi:10.1002/dad2.12633)
Supplement: Supplementary file 1 — Supporting Information [file DAD2-16-e12633-s002.pdf]

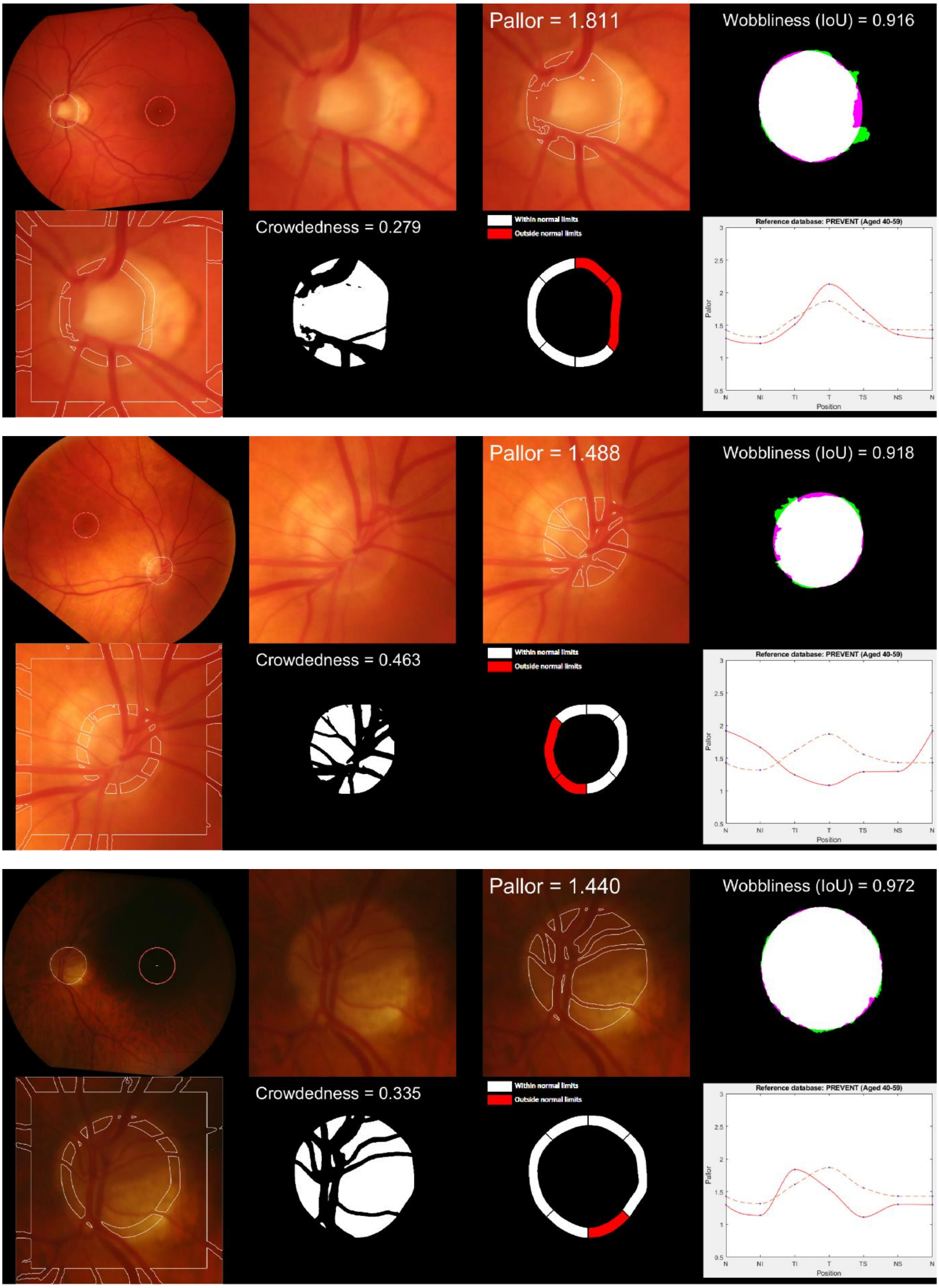

Supplemental Figure 1. Images rejected due to segmentation error (top and middle) and abnormal disc presentation (bottom). Notes: Crowdedness and Wobbliness are experimental measures that are not part of the current publication.
